# Predicting short-term suicide risk: allowing for ongoing variation in severity of intent

**DOI:** 10.1192/bjb.2020.90

**Published:** 2021-04

**Authors:** Gethin Morgan

**Keywords:** Suicide, prediction, assessment, suicidal, ideation

## Abstract

This article considers the role that assessment of suicidal ideation may have in short-term prediction of suicide. Suicide risk assessment is a multifactorial process and it is assumed that assessment of suicidal ideation is one component. Denial that suicidal ideation has any useful role in risk assessment fails to allow for the marked ongoing short-term variability in severity of intent, which is a common feature of the suicidal state of mind. It is concluded that the assessment of suicidal ideation, provided it is carried out correctly and applied appropriately, should continue to be regarded as a central component of the overall prediction process. A ‘two-take’ approach to short-term risk assessment and mitigation is proposed that takes variability in severity of intent into account and includes anticipatory treatment planning for any problems that may occur in the near future.

When a suicide occurs in one of our patients, as clinicians it is only too easy to blame ourselves even though our care had been exemplary. Nagging doubts easily linger as to whether the suicide might have been prevented if we had done this or that in a different way. It is very important in such circumstances to take a step back, accept that it can indeed be very difficult to predict suicide, let alone prevent it, and acknowledge that occasionally quite inevitably we will not succeed.^1^

However, in our day-to-day work we are probably more effective in preventing suicide than we allow ourselves to acknowledge. Such a happening goes unnoticed and is never recorded: a prevented suicide is a statistical non- event for which we get no credit. Yet no doubt it can occur. So even though the task is difficult, it is important that we should continue to scrutinise the effectiveness of our clinical skills, strive to achieve their further improvement and only discard them with caution.

This review focuses particularly on assessment of suicidal ideation, evaluating its role in the care of suicidal individuals and in predicting short-term suicide risk. Assessment of suicidal ideation is regarded as a central clinical component of the overall risk assessment process, which is of course multifactorial and has a wide clinical/social base.^2^ Long-term prediction is a highly questionable exercise and I do not consider it here.

In in-patient care, surely there can be no controversy regarding the importance of suicidal ideation: careful monitoring of any suicidal ideation that may be present is essential. Management of serious immediate risk is incomplete without minute-by-minute awareness of level of intent, and awareness of risk level should be part of any decision concerning a patient being allowed leave the ward. In the out-patient clinic evaluation of risk level, provided it can be shown to achieve an acceptable degree of reliability, should not only help us to determine the risk level at the time the patient is seen: but further it should also guide us regarding ongoing risk, thereby helping to decide the optimal length of time to the next out-patient appointment, as well as clarifying the most appropriate treatment.

## Assessment of suicidal ideation criticised

A review of several meta-analyses concerning suicide prediction reached the following conclusion: ‘None of the meta-analyses found that any individual clinical risk factor, including suicidal thoughts and behaviours, was sufficiently accurate as a basis to allocate interventions’.^3^

Further cautionary advice comes from the National Institute for Health and Care Excellence (NICE), which states in its 2011 guidelines on self-harm: ‘Do not use risk assessment tools and scales to predict future suicide or repetition of self-harm [or] to determine who should or should not be offered treatment or who should be discharged’.^4^

A number of other studies have found that the majority of patients who died by suicide had denied having suicidal ideas at the time of the final interview.^5–7^ This has been taken to mean that assessment of suicidal ideation is not to be recommended as part of suicide prediction because of its unreliability.

In the face of such a consistent body of evidence suggesting that it has no value, I will review the part that assessment of suicidal ideation might play in short-term suicide prediction. In doing so I will scrutinise the way it is used and asks whether this is appropriate.

## Assessment of suicidal ideation looked at anew

### Criticisms answered

The assessment of suicidal ideation, although it is a fundamental element of any comprehensive attempt to assess risk, often seems to be taken for granted and not described in any detail even in research literature. The procedure itself has been subjected to criticism and this needs to be answered.

There is no reason to believe that appropriate enquiry concerning the presence of suicidal ideation increases suicide risk. Nor is a person who volunteers to having suicidal ideas necessarily at low risk. Exploration of suicidal ideation is not an isolated exercise. Throughout it must be carried out in full knowledge of the total clinical picture and integrated into the whole therapeutic effort. It is a sensitive empathic reaching out to encourage sharing of distressing and often frightening ideas, as dynamic as any other form of intervention can be. It should not be a questionnaire-type Yes/No interrogation. It should involve progressive exploration of suicidal ideas themselves, their intrusiveness, frequency and acceptance as reasonable, and also of feelings of loss of control, perhaps part of delusional thinking, or of seeing suicide as the only way out of an impossible life situation. The role of ambivalence and personality traits tending towards the impulsive should never be ignored. All this requires gentle patience and warm concern. It is not intended to be a matter of focusing on a patient's weaknesses rather than strengths: it is simply a guide that can help us make things safe. At all points we need to remember that it is for the benefit of the patient and not, for example, just a way primarily of coping with our own anxieties concerning adverse sequelae if things go wrong. A confident, realistically encouraging attitude is essential: an ambivalent patient will soon pick up doubts on our part and the whole exercise can then become counterproductive. It is never appropriate as a carer to identify with a patient's despair. It needs to be recognised that aggressive behaviour can occur in parallel with significant suicide risk. Clarify any risk to others. A brusque hurried approach can leave the patient feeling stigmatised and undervalued, as well as encouraging denial of intent or even encouraging suicidal motivation. Discussing together any reasons why denial may occur and how to mitigate them, being available and offering urgent help at any time should suicidal thoughts recur, both represent a mark of respect for the patient's intrinsic worth.

Although all of this is a complex and difficult clinical task it would be a mistake to lose sight of the important positive part that discussing suicidal ideation can have in caring for suicidal individuals. To carry it out properly is comparable to having responsibility for dispensing medicines: the results of maladministration of either could be dire. So, it is reasonable to expect that each psychiatric hospital ward should ensure that all clinical staff have been well trained in assessing suicidal ideation. Appropriate privacy is important if this sensitive and complex task is to be carried out reliably.

### Allowing for variability in severity of intent

To base assessment of ongoing risk on the individual’s mental state during a single interview is clearly likely to be highly unreliable. It can mislead not because suicidal ideation is intrinsically valueless in prediction and should be discarded, but rather because such an approach makes no allowance for the marked short-lasting and ongoing variation in severity of intent that is a common feature of the suicidal state of mind. The following case series and studies illustrate this in suicidal individuals during the weeks before death.

Over a 10-year period (1968–1978), 12 deaths by suicide occurred among patients who were under my sole consultant care, either while they were resident in a single psychiatric hospital ward or within 2 months of discharge. They had all expressed suicidal ideation. I knew each of them well, day in, day out, usually over several weeks, yet in spite of my close continuing contact with them and the majority reassuring me about their safety, they ended their lives. In an attempt to understand all this further, I put all their case records together and looked at them as a whole.^8^ A half showed marked variability in severity of suicidal intent, in some even over the course of a single day, with short episodes of corresponding misleading clinical improvement in which suicidal ideas were denied. Variable contact and unresolved stress factors and impulse seemed relevant. For example, one patient happily organised a charity walk from the hospital only to take her own life the following weekend when, still apparently well, she went home on leave to be confronted with an unresolved domestic crisis.

Two later case series consisted of all psychiatric in-patients in the City of Bristol during two separate periods (1982–1984 and 1991–1993) who died by suicide either from hospital wards or within 2 months of discharge.^9,10^ In the two studies, 52 and 61% respectively showed episodes of misleading clinical improvement. Stress was a common background to relapse: 44 and 50% respectively killed themselves when clinically improved but with stress factors unresolved.

Although the number of cases in these three studies was small (12, 27 and 18 respectively) and they bridged a time span of 30 years, the findings were derived from detailed observations at ward level by clinicians responsible for the patients’ clinical care and were remarkably consistent. They confirmed that short-term variability of intent exists in a considerable proportion of suicidal patients. This reinforces the view that to judge assessment of suicidal ideation as a predictor of ongoing suicide risk merely on its efficacy when recorded in a single interview ignores a common clinical feature of the suicidal state of mind, namely its variability in severity of intent. To put it bluntly, it asks the impossible of it.

The following discussion focuses particularly on out-patient work but its principles apply to the in-patient situation too. We need to ask: In the face of such practical difficulties what can we do to make prediction of suicide more reliable so that we can rely on it over the short term until the next out-patient interview?

## An attempt to take things forward

The approach which is proposed here tries to take into account variability in severity of intent when predicting suicide risk. It is suggested that, in addition to assessing risk specific to the time of interview, any attempt at predicting ongoing short-term risk should also invariably include a projected evaluation of whether suicidal ideation is likely to recur if difficulties are encountered before the next clinical contact with the patient. This represents an attempt to ameliorate the confounding effect of ongoing variation in severity of intent. It also allows anticipatory treatment plans to be laid for any difficulties that may recur, thereby targeting therapeutic efforts more efficiently. Assessment of suicidal ideation remains a central component of the whole process. The following proposed guidance incorporates this approach with regard to follow-up of patients who have recently experienced an episode of significant suicidal feelings. It is provisional, its aim being to highlight the points made in this article and to stimulate debate regarding them. It has not been used clinically nor evaluated in any way. However, it is hoped that, after wider evaluation and any necessary amendments have been made, its principles might prove to be of practical value in taking forward the difficult task of predicting short-term suicide risk.

### Out-patient care of suicidal individuals: guidance on assessment of short-term ongoing risk

Remember that predicting ongoing risk of suicide merely on the basis of the patient's mental state at the time of a single interview can be very unreliable. Evaluation of suicidal ideation and intent should be more broadly based and cover the whole of the follow-up period. Remember that ongoing day-to-day variation in severity of intent can be marked. Always give serious consideration to any evidence of suicidal intent, expressed or otherwise. Keep in mind the possible reasons specific to each case why relapse might occur, as well as the overall risk assessment picture. An important trigger for relapse is stress, particularly stress that has previously been present and has not been resolved. Try to assess the likelihood that difficulties, stress related or otherwise, will recur before the next appointment and whether suicidal ideation will complicate them. Overall evaluation should invariably take such anticipated risk into account. Clarify what urgent help could be made available in such a forthcoming crisis, ensure that it would be acceptable to the patient, work through any hesitation expressed regarding seeking that help and review the appropriateness as well as any security provision related to prescribed medication. All this should be integral to the assessment process.

### Implementing the suggested guidance

This guidance is put forward in the hope that it might improve our ability both to predict suicide risk in the short term and to target more effectively the ongoing treatment we offer. It is no more than a care plan, but one that not only focuses on the present: it also insists on searching for potential risk at some point in the future. According to it, no assessment of ongoing suicide risk would be regarded as complete without such a ‘two-take’ approach covering the follow-up period and aimed at what amounts to a moving target. This allows plans to be laid in advance for an acceptable form of urgent help should problems recur. Predicted suicidal ideation at any level of severity without stratification and particularly ideation that is triggered by a stressful event should be taken as sufficient to indicate significant continuing risk.

As mentioned above, variability in severity of intent has been found to occur in about half of patients with suicidal ideation who proceed to suicide.^8,9,10^ In the remaining patients, environmental stress factors that presumably trigger such variability are presumably either absent or do not cause significant problems for the patient. Does the proposed guidance therefore have value only for half of patients? Not necessarily. It is possible that, in other patients, careful anticipatory enquiry might reveal a need to plan help for problems that otherwise would have remained unexpected.

From what has been presented here it is clear that the process of predicting suicide risk even in the short term is a difficult clinical exercise. Regarding someone whose stress factors have not been fully resolved, a letter to the general practitioner (GP) taking all relevant issues into consideration might read as follows:

‘Suicidal intent appears to be low or non-existent today. This is not in itself a reliable predictor of ongoing suicide risk, which could recur again for a variety of reasons relevant to the illness itself or significant stress. Our discussion today showed that such stress might well recur prior to the next appointment and the patient was anxious about having to face it. Recurrence of suicidal ideation could not be ruled out. We discussed ways in which we could offer urgent help in such circumstances and as a result the patient felt more confident about being able to get through it all. Overall, however, the predicted level of suicide risk must still be regarded as significant, requiring vigilance until I next see him/her.’

Great care should be taken before one is ever tempted to suggest that ongoing risk is totally absent in someone who has experienced suicidal ideation in the recent past. In such a situation a letter to the GP might read along the following lines:

‘The patient denied having suicidal ideas today, stress factors appear to have been resolved, family/social support has always been strong and remains so, and adverse events that might lead to relapse seem unlikely to recur during the follow-up period before I see him/her again. In spite of this reassuring picture, vigilance is required during the follow-up period. This is because relapse, especially if stress related, can occur unexpectedly in anyone who has been at risk of suicide and it is early days since he/she experienced suicidal ideation. I have discussed with him/her the availability of urgent help and as a result he/she feels confident about being able to cope until the next time we meet. For the moment, the predicted level of suicide risk must remain as uncertain.’

## Conclusions

It is hoped that the dynamic ‘two-take’ approach to predicting ongoing short-term suicide risk that is proposed here might prove to be a useful contribution to the overall risk assessment process by helping to ameliorate the ‘moving target’ problem due to varying levels of intent over short periods of time. Ongoing treatment should also be targeted more precisely as a result of its forward-looking approach. There are several other ways in which assessment of suicidal ideation can have a useful role in caring for suicidal individuals. Its value in detecting a certain group of in-patients especially vulnerable to suicide has been described elsewhere.^11^ Whatever the setting, shared knowledge of suicidal ideation can also contribute to a therapeutic alliance with the patient, promoting a readiness to discuss suicidal ideas fully and thereby helping to alienate such ideas, making defensive denial less likely. Such mutual collaboration and trust not only have therapeutic and preventive value: they can then also facilitate the process of prediction.

Given this overall picture it seems reasonable to conclude that the assessment of suicidal ideas, provided it is carried out correctly, applied appropriately and always used within the wider context of risk assessment as a whole, can play a valued part in the overall care and prediction of risk in suicidal individuals. Surely it is here to stay.

## Data Availability

Data availability is not applicable to this article as no new data were created or analysed in this study.

## About the author

Gethin Morgan, MD Cantab, FRCP, FRCPsych, DPM is Emeritus Norah Cooke Hurle Professor of Mental Health in the Department of Psychiatry at the University of Bristol, UK.
